# Centrally Administered Ghrelin Acutely Influences Food Choice in Rodents

**DOI:** 10.1371/journal.pone.0149456

**Published:** 2016-02-29

**Authors:** Erik Schéle, Tina Bake, Cristina Rabasa, Suzanne L. Dickson

**Affiliations:** Institute of Neuroscience and Physiology, The Sahlgrenska Academy at the University of Gothenburg, Gothenburg, Sweden; University of Santiago de Compostela School of Medicine - CIMUS, SPAIN

## Abstract

We sought to determine whether the orexigenic hormone, ghrelin, is involved in the intrinsic regulation of food choice in rats. Ghrelin would seem suited to serve such a role given that it signals hunger information from the stomach to brain areas important for feeding control, including the hypothalamus and reward system (e.g. ventral tegmental area, VTA). Thus, in rats offered a choice of palatable foods (sucrose pellets and lard) superimposed on regular chow for 2 weeks, we explored whether acute central delivery of ghrelin (intracerebroventricular (ICV) or intra-VTA) is able to redirect their dietary choice. The major unexpected finding is that, in rats with high baseline lard intake, acute ICV ghrelin injection increased their chow intake over 3-fold, relative to vehicle-injected controls, measured at both 3 hr and 6 hr after injection. Similar effects were observed when ghrelin was delivered to the VTA, thereby identifying the VTA as a likely contributing neurobiological substrate for these effects. We also explored food choice after an overnight fast, when endogenous ghrelin levels are elevated, and found similar effects of dietary choice to those described for ghrelin. These effects of fasting on food choice were suppressed in models of suppressed ghrelin signaling (i.e. peripheral injection of a ghrelin receptor antagonist to rats and ghrelin receptor (GHSR) knock-out mice), implicating a role for endogenous ghrelin in the changes in food choice that occur after an overnight fast. Thus, in line with its role as a gut-brain hunger hormone, ghrelin appears to be able to acutely alter food choice, with notable effects to promote “healthy” chow intake, and identify the VTA as a likely contributing neurobiological substrate for these effects.

## Introduction

The neurobiology of food choice remains a less chartered landscape in obesity research, partly reflecting the large number of intrinsic and environmental factors that guide it [1]. While dietary choice is clearly influenced by an individual’s food preference [2], such preferences can be over-ridden. A particularly striking example is the dramatic change in food preference and dietary choice behavior that occur in obese individuals that have undergone gastric bypass (a bariatric weight loss surgery)[3]; these changes appear not to be secondary to physical (eg restrictive or malabsorptive) consequences of the surgery but must rather involve responses of unconscious intrinsic physiological, control systems.

In the present study, we sought to determine whether ghrelin, a circulating stomach-derived hormone [4], participates in the intrinsic regulation of food choice behavior in rats. Ghrelin would seem a good candidate to steer dietary choice given that it appears to signal hunger information from the empty stomach [5] to brain areas important for feeding control, including areas linked to food reward/motivation [6, 7]. In man, ghrelin secretion is especially marked before mealtimes [8] and circulating levels correlate strongly with self-reported feelings of hunger [5]. Given that ghrelin administration not only promotes food intake but also increases hunger scores in healthy volunteers [9], it is generally assumed that ghrelin confers hunger information to the brain. Studies in rodents demonstrate that ghrelin is able to orchestrate a number of behavioral responses that extend beyond food intake to include food reward [10], food-anticipatory [11, 12] and food-motivated behaviors [13–15]. Importantly, at the level of the ventral tegmental area (VTA, an area important for reward), ghrelin is able to drive food-motivated behavior and food intake [14, 16], yet we do not know if ghrelin action at this site has consequences for food choice.

The aim of the present study was therefore to explore the impact of acute delivery of ghrelin on food choice behavior in rats. To study food choice behavior, rats were fed an obesogenic food choice diet comprising chow, sucrose pellets and lard (saturated animal fat) and, we explored the effects of acute delivery of ghrelin to the brain ventricles or to the VTA on food choice behavior. Given that ghrelin operates as a circulating hunger hormone, with high endogenous levels during fasting [17, 18], we sought to determine the impact of an overnight fast of food choice behavior and whether this is altered in models of suppressed ghrelin signaling.

## Materials and Methods

### Animals

Adult male Sprague-Dawley rats (Charles River Laboratories, Wilmington, MA, USA) were used in all injection experiments. In the brain administration experiments the body weight of the rats at the time of surgery was 250–300 gram. During the injection period they weighed 370–430 gram. Male GHSR knock-out (KO) mice (Deltagen, San Mateo, CA, USA) on a C57bl6 background, and their wild-type (WT) littermates were also used in the study. Animals were kept under standardized non-barrier condition on a 12/12 hour light/dark cycle at 20°C and 50% humidity. On arrival in the animal facility, animals had *ad libitum* access to standard maintenance chow (Teklad diet 2016, Harlan Laboratories, Cambridgeshire, UK). Rats were switched to a choice diet (see below) at 14 days prior to the experimental studies. Mice were switched to the choice at 8 weeks of age and remained on it for 2 weeks prior to the experimental study. Water was available *ab libitum* at all times. The animal procedures were approved by the local ethics committee for animal care in Gothenburg, Sweden (Göteborgs djurförsöksetiska nämnd; permit number 27–2015 and 28–2015), and were conducted in accordance with guidelines.

### Guide cannula implantation

The rats were anaesthetized with a combination of Rompun^®^ vet. 10 mg/kg (Bayer, Leverkusen, Germany) and Ketaminol^®^ vet. 75 mg/kg (Intervet, Boxmeer, Netherlands) and placed in a stereotaxic frame. The skull bone was exposed and the skull sutures were identified. Holes for guide cannulae and anchoring screws were drilled through the skull. A 26 gauge cannula was positioned according to stereotactic coordinates and fixed in place with anchoring screws and dental cement. For guide cannula placement in the lateral ventricle, the following coordinates were used: 0.9 mm posterior to bregma, ± 1.6 mm lateral to the midline and 2.5 mm ventral of the skull surface. For VTA unilateral cannula placement, the following coordinates were used: 5.7 mm posterior to bregma, ± 0.75 mm lateral to the midline and 6.5 mm ventral of the skull surface. The coordinates correspond to 2 mm dorsal to the target brain area, as injection cannulae were 2 mm longer than the guide cannulae.

After surgery the rats received an analgesic (Rimadyl^®^ vet. 5 mg/kg, Orion Pharma Animal Health, Sollentuna, Sweden) and were singly housed and allowed to recover for at least 4 days. To validate cannula placement in the lateral ventricle, conscious rats were injected with angiotensin II for which correct placement is indicated by a dipsogenic (immediate water drinking) response [19]. The week after the last food choice measurement the anesthetized (Isofluran, Baxter, Deerfield, IL, USA) rats were euthanized by decapitation. Correct cannula placement in the VTA was confirmed post mortem by visualization of injected ink (Chicago sky blue 6B) at a volume of 1 μl.

### Food choice measurements

Mice or individually housed rats were introduced to a free choice *ab libitum* feeding paradigm consisting of standard maintenance chow pellets, 1 g sucrose pellets (Sandown Scientific, Hampton, UK) and lard (saturated animal fat; Dragsbæk, Thisted, Denmark). The distribution in energy between macronutrients in the chow diet was 66% carbohydrate, 22% protein and 12% fat. The animals were kept on this free choice diet throughout all experiments, except during the overnight fast. During the 14 day baseline period, 24 hr food intake was measured on a regular basis. For the central injection studies in rats, on the injection day, food intake was measured at 3 hr, 6 hr and 24 hr post-injection. All injections were performed early in the light phase between 9.00 and 10.00 am.

### Central administration (ICV and intra-VTA) of ghrelin

In both the ICV study and the intra-VTA study injections were performed according to a cross-over balanced experimental design, in which all rats at one point received an injection of either ghrelin or artificial cerebrospinal fluid (aCSF; Tocris, Bristol, U.K) separated with one day wash out period. This corresponds to days 15 and 17 of exposure to the choice diet. All the injections were performed over a 1 min period using a 10 μl Hamilton syringe coupled to an injector needle via vinyl tubing. After injection the injector was kept in place for approximately 2 min to ensure full diffusion from the injector tip. The injected volume was 2 μl into the lateral ventricle, and 1 μl unilaterally into the VTA. Ghrelin (1465; Tocris, Bristol, UK) and NPY (H-6375; Bachem, Bubendorf, Switzerland) were dissolved in aCSF. Ghrelin was injected at a dose of 2 μg ICV, and 1 μg intra-VTA, doses that previously has been shown to induce a feeding response in rats [16, 20]. For intra-VTA injection, we used coordinates that correspond to the published distribution of the ghrelin receptor (GHS-R1A)[21] and used a 1μl delivery volume in order to ensure that we reached the entire VTA with the injected substance.

### Peripheral administration of a ghrelin receptor (GHSR-1A) antagonist to fasted rats

Prior to injection, all rats had undergone mock injections to familiarize them with the intraperitoneal (i.p.) injection procedure and hence, limit stress associated with it. Injections were performed according to a cross-over balanced experimental design, in which all rats (n = 24) at one point received either the ghrelin receptor antagonist, JMV2959 (6 mg/kg; gift from AeternaZentaris GMBH, Frankfurt, Germany) or an equal volume (0.2 ml) saline vehicle separated by a one day wash out period. The rats had been on the choice diet for 2 weeks prior to injection. Rats were fasted overnight prior to each injection, which increases endogenous ghrelin levels [17, 18]. Immediately after injection the rats were reintroduced to the food choice paradigm (chow, sucrose pellets and lard) and food intake monitored as before.

### Statistical analysis

The data were analyzed using a paired student’s t-test unless otherwise stated. Repeated measures analysis of variance (rANOVA) was used to compare three within subject conditions in the intra-peritoneal injection experiment. In this case, the three conditions (factors) were; baseline calorie intake, calorie intake after fasting, calorie intake after fasting plus JMV2959 treatment. One-way ANOVA was used for the GHSR-KO mouse study. P < 0.05 was considered statistically significant.

## Results

### ICV injection of ghrelin increases chow and lard, but not sucrose intake

**Fig 1:**
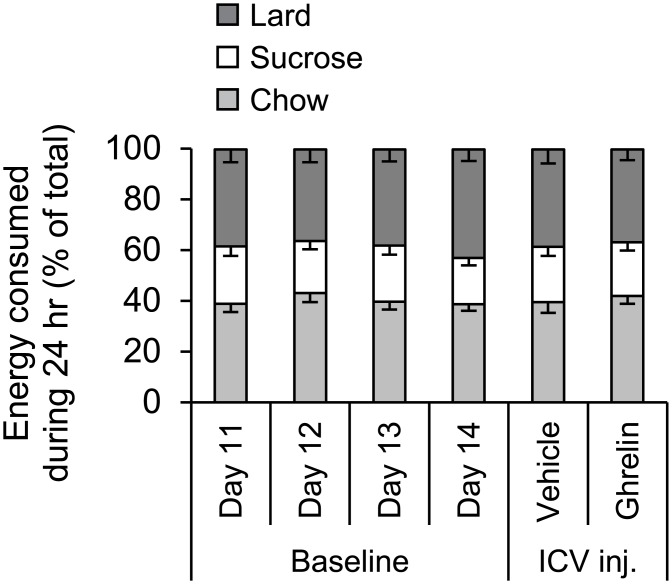
Stability of daily food selection. Stability of daily (24 hr) food selection in rats (n = 18) offered an *ad libitum* free choice diet of normal chow, 1 g sucrose pellets and lard (a saturated animal fat). Data are shown for the last 4 baseline days (day 11–14) prior to ICV injection, and for the injection days in which 2 μg ghrelin or aCSF were administered. The data are expressed as mean ± SEM kcal consumed as % of daily total kcal intake.

Rats (n = 18) were given a free choice diet comprising chow, sucrose pellets and lard over a time period of 14 days. During the last 4 days of the baseline period, 24 hr energy intake and food choice was stable (Fig 1), and almost identical to that reported in a previous study [22].

**Fig 2:**
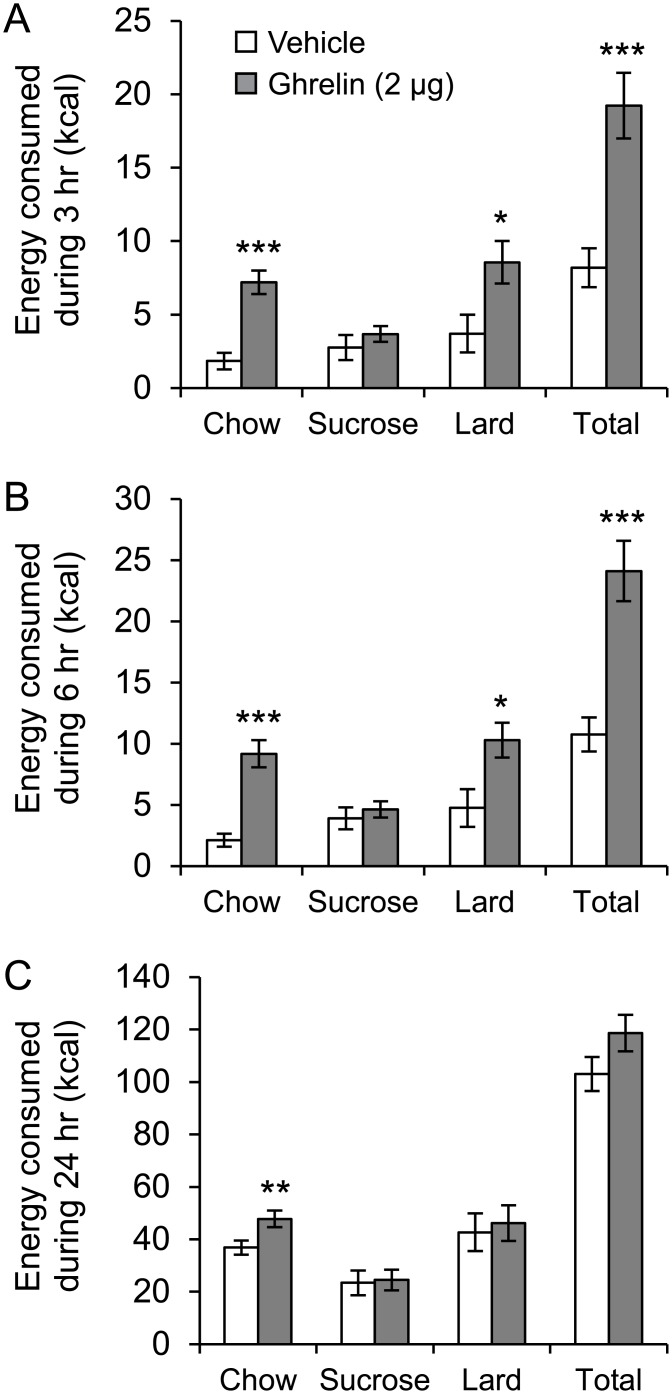
Impact of ICV ghrelin on dietary choice. Impact of acute intracerebroventricular (ICV) injection of ghrelin on total energy intake and dietary choice during (A) 3 hr, (B) 6 hr and (C) 24 hr, in rats (n = 18) that had been *ad libitum* fed a choice diet comprising normal chow, 1 g sucrose pellets and lard (saturated animal fat) over the previous 14 days. All rats received ghrelin and vehicle solution in a cross over design with one day washout between injections. *P<0.05, **P<0.01, ***P<0.001.

Following this 14 day baseline period, rats were injected ICV and cumulative food intake was measured at 3 hr, 6 hr and 24 hr after injection (Fig 2). By 3 hr after ICV injection, total energy intake was 19 ± 2 kcal for ghrelin delivery compared to 8 ± 1 kcal after vehicle, an over 2 fold increase (P<0.001). This orexigenic effect was still evident at 6 hr post-injection by which time total energy intake was 24 ± 3 kcal for ghrelin treatment compared to 11 ± 1 kcal for vehicle treatment (P<0.001). As expected [23], cumulative energy intake over a 24 hr period was not increased by ICV ghrelin relative to aCSF vehicle (Fig 2) and resembled that of the baseline period (Fig 1).

The effects of ghrelin to increase energy intake were not evenly distributed between the three foods offered. Perhaps the most unexpected result was that ICV ghrelin induced a marked significant increase in chow intake relative to vehicle administration at all three time points. Ghrelin injection increased cumulative chow intake by >3 fold (P<0.001) compared to vehicle injection measured at both 3 hr and 6 hr post injection. After 24 hr, despite no differences in overall energy intake, the ghrelin-induced increase in kcal from chow was still evident even although not as marked, now 30% larger compared to vehicle treatment (P<0.01). Ghrelin also increased cumulative lard intake by 2 fold (P<0.05) compared to vehicle measured at both 3 hr and 6 hr post injection. By 24 hr after injection, lard intake did not differ significantly between the ghrelin and vehicle treatments. Interestingly, there were no changes in cumulative sucrose intake at any of the three time points after injections (Fig 2).

### Intra-VTA injection of ghrelin increases chow and lard intake, but not sucrose intake

On day 14 of feeding the rats (n = 17) the free choice diet, baseline chow intake was 38 ± 3 kcal sucrose intake was 16 ± 4 kcal and lard intake was 65 ± 6 kcal (21%, 13% and 54% of total daily kcal consumed, respectively). Thus, at the time of injection, a large proportion of total energy intake came from lard.

**Fig 3:**
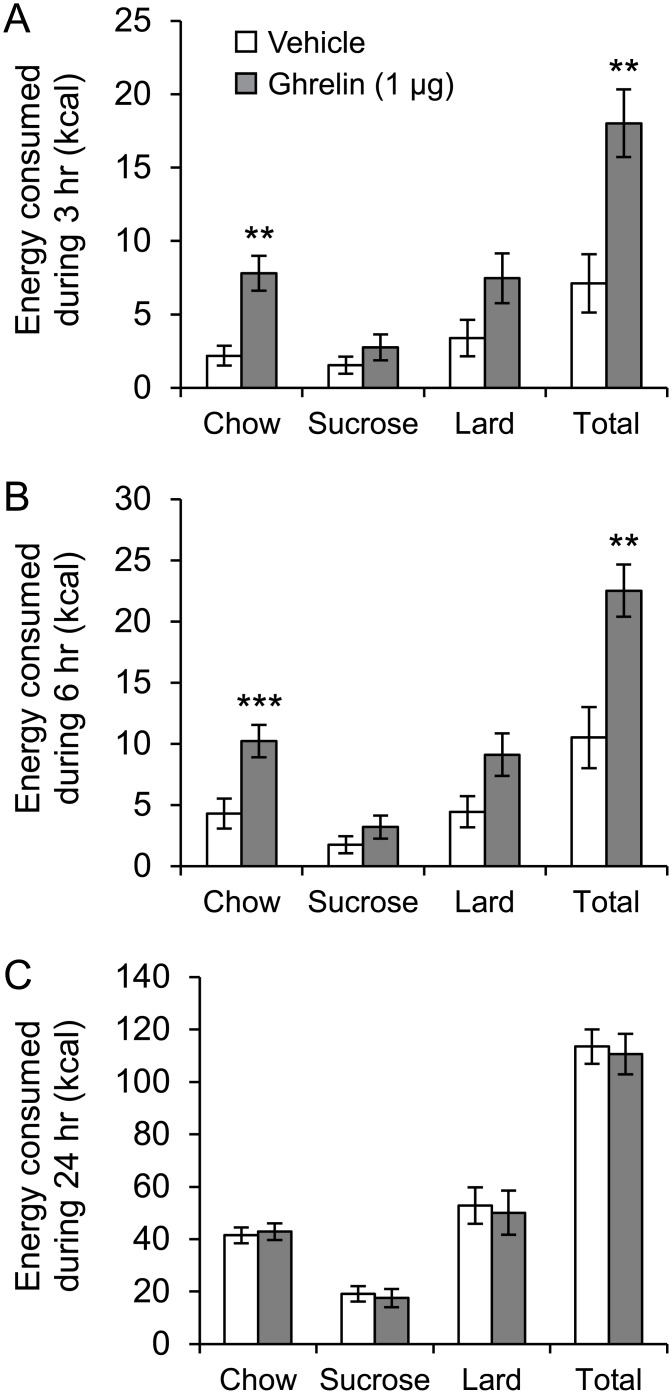
Impact of intra-VTA ghrelin on dietary choice. Impact of acute intra-VTA (ventral tegmental area) injection of ghrelin on total energy intake and dietary choice during (A) 3 hr, (B) 6 hr and (C) 24 hr, in rats (n = 17) that had been *ad libitum* fed a choice diet comprising normal chow, 1 g sucrose pellets and lard (saturated animal fat) over the previous 14 days. All rats received ghrelin and vehicle solution in a cross over design with one day washout between injections. **P<0.01, ***P<0.001.

Intra-VTA ghrelin increased total kcal intake by over 2 fold (P<0.01) compared to vehicle injection measured at both 3 hr and 6 hr post-injection. By the 3 hr time point, total cumulative energy intake was 18 ± 2 kcal for ghrelin delivery compared to 7 ± 2 kcal for vehicle administration, and by the 6 hr time point cumulative intake was 23 ± 2 kcal for ghrelin and 10 ± 3 kcal for vehicle. At 24 hr post-injection, there were no differences in cumulative food intake between ghrelin and vehicle treatments. Similar to ICV ghrelin treatment, intra-VTA ghrelin administration induced more than a 3 fold increase in chow intake compared to vehicle measured at both 3 hr (P<0.01) and 6 hr (P<0.001) post-injection, while there were no significant changes in sucrose or lard intake at any time point. However, there was a tendency towards increased lard intake after ghrelin treatment compared to vehicle at 6 hr post injection (P = 0.055) (Fig 3).

### Chow and lard intake were increased by fasting and reverted by peripheral treatment with a ghrelin receptor antagonist

On day 14 of exposure to the free choice diet, the baseline energy intake was 55 ± 1 kcal from chow, 18 ± 3 kcal from sucrose and 32 ± 4 kcal from lard (52%, 17% and 30% of total kcal consumed in 24 hr, respectively). Thus, in this cohort, lard intake was a large energy source at baseline, as before, however the intake of chow was higher than in the previous cohorts.

**Fig 4:**
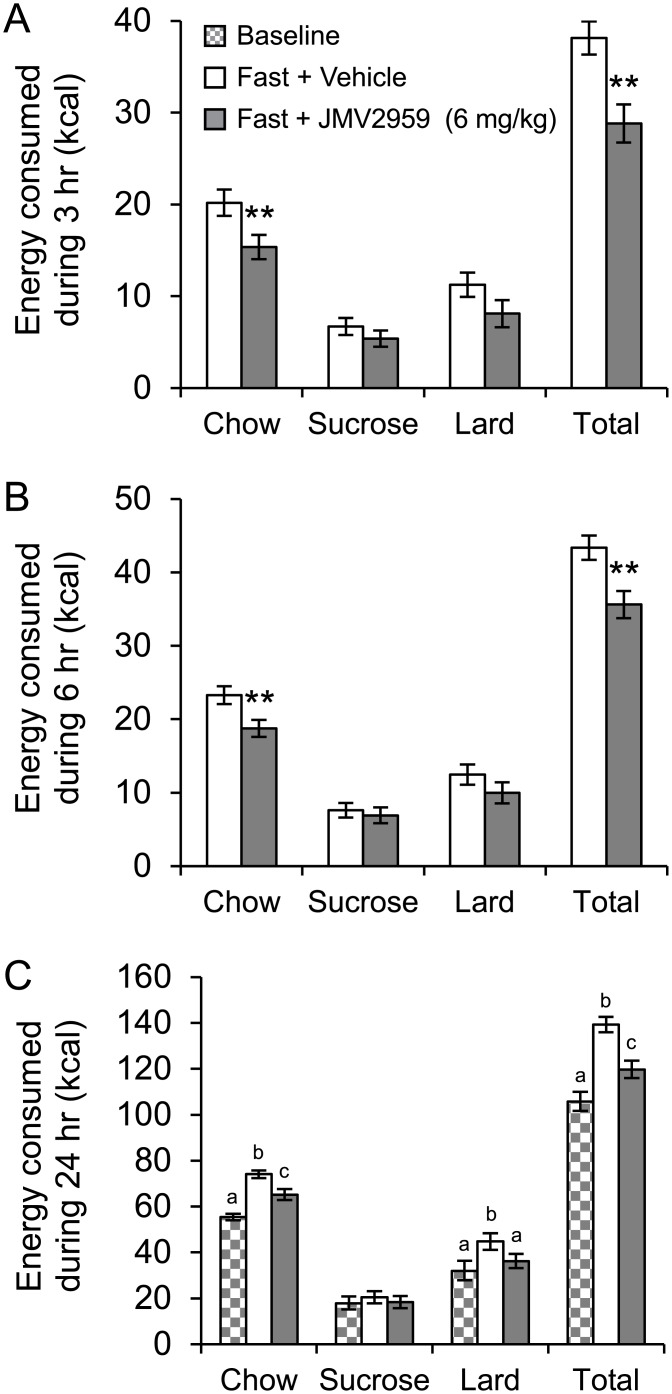
Impact of fasting and peripherally administered ghrelin receptor antagonist on dietary choice. Impact of acute intraperitoneal injection of a ghrelin receptor antagonist on fasting-induced changes in total energy intake and dietary choice measured during (A) 3 hr, (B) 6 hr and (C) 24 hr after the end of an overnight fast, in rats (n = 24) that had been *ad libitum* fed a choice diet comprising normal chow, 1 g sucrose pellets and lard (saturated animal fat) for 14 days prior to fast. All rats received the antagonist and saline solution in a cross over design with one day washout between injections. In panels A and B; **P<0.01. In panel C, the letters indicate significant differences (rANOVA, P<0.01).

After 14 days on the choice diet, the rats (n = 24) were fasted overnight after which they received an i.p. injection of a ghrelin antagonist or saline vehicle solution (in a balanced cross-over design). After reintroduction to the food choice paradigm, the effects of fasting on food choice were evident from the vehicle-treated group: total 24 hr energy intake was increased by 24% (P<0.001) compared to baseline (day 14). Chow and lard intake increased significantly by 26% (P<0.001) and by 29% (P<0.001) respectively compared to baseline (day 14), while sucrose intake remained unchanged. JMV2959 treatment to overnight fasted rats, significantly decreased food intake acutely during 3 hr by 26% (P<0.01) and during 6 hr by 16% (P<0.01), as well as during 24 hr by 14% (P<0.001) compared to saline administration. Acutely, during 3 hr and 6 hr JMV2959 decreased chow intake by 25% (P<0.01) and by 22% (P<0.01) respectively compared to saline administration, which solely accounted for the total decrease in energy intake observed. There was a tendency towards a decrease in lard intake during 6 hr (P = 0.052) by JMV2959 treatment. During 24 hr, chow intake was still decreased, now by 12% (P<0.001) by JMV2959 treatment compared to saline. In addition, lard intake during 24 hr was significantly decreased by 20% (P<0.001) compared to saline administration, and was reduced to baseline levels. Sucrose intake was unaffected by JMV2959 treatment at all time points (Fig 4).

### Depletion of ghrelin receptor in GHSR-KO mice decreased 24 hr chow intake after an overnight fast

**Fig 5:**
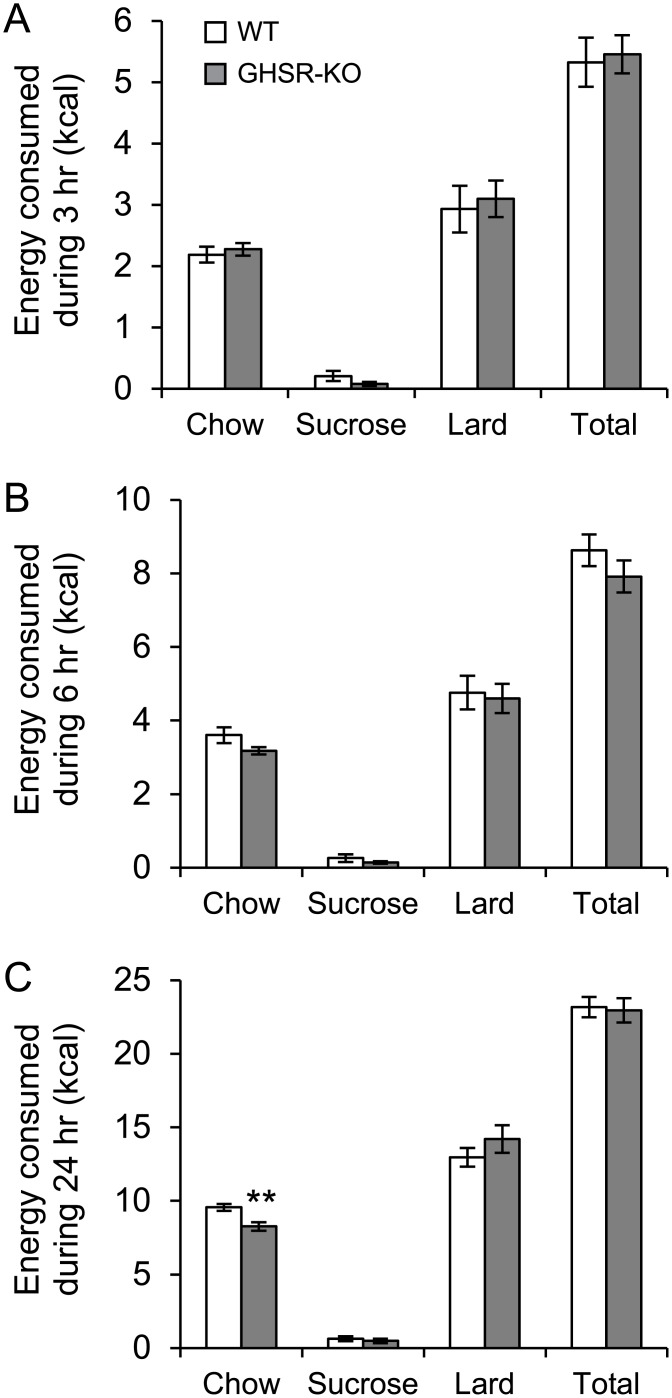
Impact of ghrelin receptor knockout on dietary choice. Total energy intake and dietary choice were measured during (A) 3 hr, (B) 6 hr and (C) 24 hr after the end of an overnight fast, in ghrelin receptor knockout mice (GHSR-KO) and wild-type (WT) controls that had been *ad libitum* fed a choice diet comprising normal chow, 1 g sucrose pellets and lard (saturated animal fat) for 14 days prior to the overnight fast. **P<0.01 (one-way ANOVA).

After 14 days on the choice diet, GHSR-KO mice (n = 9) and their littermate WT controls (n = 8) were fasted overnight. During 24 hr after reintroduction to the food choice paradigm, chow intake was significantly decreased by 14% (P<0.01) in the GHSR-KO compared to WT. Twenty-four hour total food intake as well as sucrose and lard intake after the overnight fast did not differ between GHSR-KO and WT mice. During 3 hr and 6 hr after the reintroduction of the food choice paradigm there were no difference in total food intake or in either chow, sucrose or lard intake between GHSR-KO and WT mice (Fig 5).

## Discussion

Here we demonstrate an unexpected effect of acute central injection of ghrelin, an orexigenic and reward-promoting hormone, to cause an acute >3 fold increase in chow intake in rats offered an *ad libitum* free choice diet comprising two palatable choices (lard, a saturated animal fat, and sucrose pellets) superimposed on regular chow. This effect is particularly striking because, at the time of ghrelin administration, the rats had been on this choice diet for 2 weeks, and were consuming a considerably large proportion of their caloric intake from lard. The effect of ghrelin to increase chow intake was also observed after intra-VTA ghrelin delivery, indicating that this brain target for ghrelin is a likely neurobiological substrate for these effects. In addition, we demonstrate that, after an overnight fast, when endogenous ghrelin levels are elevated [17, 18], food selection in this free choice paradigm was rather similar to that induced by ghrelin injection. Endogenous ghrelin signaling is implicated in these effects of fasting to redirect food choice, as they were largely reversed by peripheral delivery of a ghrelin antagonist. In line with this, 24 hr chow intake was lower in ghrelin receptor (GHSR) knockout mice than in wild-type mice after an overnight fast, while neither lard nor sucrose were affected. Intriguingly, in the free choice paradigm, neither ghrelin (ICV or intra-VTA) nor suppression of ghrelin signaling (ghrelin antagonist administered peripherally or GHSR-KO) changed the amount of sucrose pellets consumed suggesting that sucrose intake is ghrelin-independent.

As expected for an orexigenic hormone [20], upon central ghrelin treatment (ICV or intra-VTA), there was an acute increase in total energy intake. Given that ghrelin has been shown to increase motivated behavior for fat [15] and sucrose [13, 14], by targeting reward areas, it was our expectation that ghrelin would amplify the intake of palatable foods and that chow intake would either remain constant or decrease. The most surprising finding, therefore, concerned the chow intake that was substantially increased by ghrelin injection (ICV or intra-VTA). In the ICV ghrelin injected group (but not the intra-VTA group), lard intake also increased but there was no effect in any injection study of ghrelin on sucrose intake, which remained remarkably stable throughout. These observations are, at least in part, supported by a previous study showing that under a free choice situation of chow and liquid sucrose solution, ICV ghrelin administration acutely increased chow intake without impacting on sucrose solution intake [24]. It was also interesting to note that by the 24 hr time point after ghrelin injection, when total caloric intake did not differ from control vehicle injection, chow intake was still elevated. It will be interesting to discover why rats start to eat more chow diet under the influence of ghrelin and it is tempting to offer an explanation that includes a physiological role for ghrelin to redirect food choices in favor of options with the best nutritional value.

Previous studies exploring the effects of ghrelin on food choice are limited and involve very different dietary/preference models to the one used here and only involve 2-choice models or rats that differ in their baseline preference. For example, one study reported that ICV ghrelin preferentially enhanced fat consumption over carbohydrates in both high fat- and high carbohydrate-preferring rats [25]. One reason why we have been able to capture the effect of ghrelin to promote chow intake could be the food choice diet paradigm used; this paradigm is very similar to the well-documented high fat—high sucrose (HFHS) diet that is obesogenic [22], and which has been shown to increase motivated behavior for food (assessed in a progressive ratio lever-pressing for sucrose task) [26]. The only modification is that we used sucrose pellets instead of liquid sucrose, a modification which did not affect baseline daily caloric intake from sucrose of about 20 kcal/day.

The stability of sucrose intake of about 20 kcal/day in our food choice paradigm is also potentially rather interesting. Importantly, it seems unlikely that 20 kcal/day represents a ceiling on sugar intake as it has been demonstrated that rats are able to consume over 30 kcal of sucrose per day in a paradigm in which sucrose was offered as the only supplement to regular chow. Moreover, the observed stability of sucrose intake confirm previous findings that offering rats the combination diet of chow, sucrose and lard appears to limit sucrose intake [22]. When sucrose is the only food offered, ghrelin promotes its consumption [24] and rats will even show motivated behavior for it [13]. However, when offered together with chow [24] or in a combination with lard (the present study), sucrose is clearly not the preferred food and ghrelin does not appear to increase its selection. Arguably, it may seem unlikely the ghrelin drives chow intake because of the carbohydrate content, given that it did not increase intake of sucrose. It may be that ghrelin promotes chow intake because of some other component (e.g. protein, fiber or even vitamins and minerals) or because the carbohydrates in chow are preferred over sucrose.

We hypothesized that a likely neural substrate for ghrelin to alter food choice is the VTA to nucleus accumbens (NAcc) dopamine pathway that is involved in motivation for natural rewards (e.g. food) as well as addictive drugs. Previous studies have shown that ghrelin activates this projection: (1) ghrelin causes NAcc dopamine release [27], activates VTA neurons [28] and that VTA delivery of ghrelin induces food intake [10, 29] and increases motivation for sucrose food rewards [14], effects involving D1/D2 dopamine signaling in the NAcc [16]. In relation to food choice, one study explored the impact of chronic VTA delivery of ghrelin or a ghrelin antagonist in rats offered a choice of diets rich in fat, carbohydrate or protein. In this feeding paradigm, rats developed a natural preference for the high fat diet and, while chronic VTA ghrelin infusion over 14 days had no effect on intake of any diet, infusion of a ghrelin antagonist selectively attenuated their intake of the preferred high fat diet [30]. In the present study, we chose acute VTA delivery of ghrelin in the chow-sucrose-lard choice paradigm and found that ghrelin delivery at this site is able to guide dietary choice towards chow. Thus, we demonstrate this effect of ghrelin to acutely increase chow intake in two cohorts of rats involving two different routes of administration (ICV as well as intra-VTA) and identify the VTA as a likely neural substrate that contributes to these effects.

The question arises as to how ghrelin action, that includes direct effects at the level of the VTA, can promote motivated (reward) behavior for palatable foods [13–15] and yet redirect food choice in favor of chow. These are not mutually exclusive constructs, however. Studies to date exploring ghrelin’s effects on food motivation offer specific single foods that are thought to have a high reward value (e.g. 45 mg sucrose pellets [13, 14, 16], 45 mg chocolate pellets [30] or 20 mg high fat diet pellets [15]). If we view ghrelin as a hunger signal, it may be that ghrelin action at the level of the VTA can increase the motivational reward value of all foods, including chow and bland foods. What we can conclude, however, is that food motivation experiments do not predict food choice behavior for specific foods that is under the control of ghrelin.

Taste and flavor perception are key drivers of the development of food preference and ultimately food choice [31]. Thus, it is possible that the effect of ghrelin on food choice is are influenced by its effect on flavor perception. Indeed, peripheral administration of ghrelin to mice has been shown to increase the intake of sweet fluids regardless of their caloric content [32]. On the other hand, food deprived rats, with possible high ghrelin levels, prefer oily flavor over sweet taste compared with non-deprived rats that preferred sweet taste in a two-bottle choice test situation [33]. Here, we did not investigate the influence of taste over food choice. However, our observed stability in sucrose consumption may indicate that sweet flavor perception was not influenced by ghrelin. It would be difficult to completely rule out the possibility that fasting influences fatty flavor perception and hence contribute to the increased lard intake after an overnight fast.

Our data support a role for endogenous ghrelin in guiding dietary choice in a manner similar to that induced by brain ghrelin delivery. We demonstrated that fasting, known to increase endogenous circulating ghrelin levels [17, 18], also increases chow and lard intake in rats, effects that could be largely prevented by peripheral delivery of a ghrelin receptor antagonist. In line with this, ghrelin receptor (GHSR) knockout mice had reduced 24 hr chow intake (without any effect on sucrose or lard) after an overnight fast. However, during 3hr and 6 hr after the reintroduction to food, GHSR mice had similar feeding pattern as the WT controls, and the effect on food choice seem to be delayed in comparison to the administration of ghrelin receptor antagonist. This delay could be a result of compensatory mechanisms developed in these mice with chronic unfunctional ghrelin signaling. Surprisingly little is documented about the effects of short term fasting on food choice in rodents. One study suggested that overnight fasting increased preference for fat (in this case vegetable shortening) over carbohydrate (corn starch and powdered sugar) and protein (casein) [34]. Thus, our data implicate ghrelin in fasting-induced changes in dietary choice, consistent with its suggested role as a circulating hunger hormone.

In conclusion, we establish a role for the central ghrelin signaling system in the intrinsic regulation of food choice behavior. Given that ghrelin has emerged as a gut-brain reward signal that increases motivated behavior for palatable foods [13–15], we were surprised to discover that, in a food choice situation (comprising palatable foods in addition to chow), ghrelin acutely promoted a 3 fold increase in the intake of regular chow. Our data also implicate endogenous ghrelin signaling in the effects of fasting to redirect dietary choice behavior in favour of “healthier” chow. Finally, our data identifying the VTA as a likely substrate for these effects, suggest that ghrelin action at reward-linked brain areas is relevant not only for behaviours linked to palatable food intake but also for the promotion and selection of regular healthy foods.
